# Network Pharmacology and Molecular Docking-Based Strategy to Investigate the Multitarget Mechanisms of Shenqi Yizhi Granule on Alzheimer's Disease

**DOI:** 10.1155/2022/8032036

**Published:** 2022-04-30

**Authors:** Linshuang Wang, Xiaoyu Xu, Zikang Wang, Qian Chen, Xiaodie Wei, Jingfan Xue, Zhanjun Zhang, Miao Wang, Yanping Li, Junying Zhang, Dongfeng Wei

**Affiliations:** ^1^Institute of Basic Research in Clinical Medicine, China Academy of Chinese Medical Sciences, Beijing 100700, China; ^2^College of Traditional Chinese Medicine, Chongqing Medical University, Chongqing 400016, China; ^3^Department of Rheumatology, Chongqing Hospital of Traditional Chinese Medicine, Chongqing 400021, China; ^4^Xi'an Medical University, Xi'an 710000, China; ^5^Huaiyin Normal University, Huai'an 223001, China; ^6^State Key Laboratory of Cognitive Neuroscience and Learning & IDG/McGovern Institute for Brain Research, Beijing Normal University, Beijing 100875, China; ^7^BABRI Centre, Beijing Normal University, Beijing 100875, China

## Abstract

**Background:**

Traditional Chinese herbal medicine draws more attention to explore an effective therapeutic strategy for Alzheimer's disease (AD). Shenqi Yizhi granule (SQYG), a Chinese herbal recipe, has been applied to ameliorate cognitive impairment in mild-to-moderate AD patients. However, the overall molecular mechanism of SQYG in treating AD has not been clarified.

**Objective:**

This study aimed to investigate the molecular mechanism of SQYG on AD using an integration strategy of network pharmacology and molecular docking.

**Methods:**

The active compounds of SQYG and common targets between SQYG and AD were screened from databases. The herb-compound network, compound-target network, and protein-protein interaction network were constructed. The enrichment analysis of common targets and molecular docking were performed.

**Results:**

816 compounds and 307 common targets between SQYG and AD were screened. KEGG analysis revealed that common targets were mainly enriched in lipid metabolism, metal ion metabolism, IL-17 signaling pathway, GABA receptor signaling, and neuroactive ligand-receptor interaction. Molecular docking analysis showed high binding affinity between ginsenoside Rg1 and A*β*_1–42_, tanshinone IIA and BACE1, baicalin, and AchE.

**Conclusions:**

The therapeutic mechanisms of SQYG on AD were associated with regulating lipid metabolism, metal ion metabolism, IL-17 signaling pathway, and GABA receptor signaling. Ginsenoside Rg1, tanshinone IIA, baicalin, astragaloside IV, and folic acid may play an important role in AD treatment.

## 1. Introduction

Alzheimer's disease (AD) is a neurodegenerative disorder characterized by progressive loss of cognitive function, thought slowness, behavioral abnormalities, and irreversible weakness of daily living [1]. A total of 30 million AD patients worldwide have been recorded now. Without advances in effective therapeutics for dementia, the number of AD patients would rise to 100 million worldwide by 2050 [2]. Extracellular *β*-amyloid (A*β*) plaques and intraneuronal neurofibrillary tangles caused by phosphorylated tau proteins have been characterized as key pathologic features of AD [3]. In addition, a variety of factors contributed to the development of AD, including glucose metabolism, mitochondrial dysfunction, synaptic transmission failure, oxidative stress, and cell apoptosis [4–7].

Although AD has been extensively studied for many years, its therapies are still lacking due to the complex pathology. Traditional Chinese medicine (TCM) has a long history of widely applications in Asia [8]. TCM showed remarkable efficacy in preventing and treating neurodegenerative illnesses based on its multicomponent and multitarget effects, which may bring novel therapy options for AD [9]. Shenqi Yizhi granule (SQYG) is a Chinese herbal recipe for AD treatment based on the theory of TCM. SQYG consists of *Panax ginseng* C.A.Mey, *Astragalus membranaceus* (Fisch.) Bunge, *Scutellaria baicalensis* Georgi, *Salvia miltiorrhiza* Bunge, and *Alisma plantago-aquatica* L with a ratio of 2 : 4 : 3 : 3 : 2. The main active compounds of SQYG included ginsenoside Rb1, ginsenoside Rg1, ginsenoside Rd, baicalin, cryptotanshinone, and tanshinone IIA [10]. These active compounds have a wide range of activities, including neuron cells protection, synaptoprotective effect, antioxidation, promoting energy metabolism, anti-inflammation, lipid composition regulation, and metal ion homeostasis, which are all key points in AD therapy [11, 12]. SQYG has been used in clinical treatment of mild-to-moderate dementia in China, exerting a potent role in neuroprotection [13]. Previous studies have shown that SQYG ameliorated the cognitive impairments in APP/PS1 mice by inhibiting neuronal loss, soluble A*β* deposition, tau hyperphosphorylation, and inflammation [14]. The hippocampus of 5XFAD transgenic mice treated with SQYG presented fewer A*β* deposits and reduced A*β*_1–42_ levels [10]. The neuroprotective mechanisms of SQYG on the hippocampus of 5XFAD mice were related to modulation of multiple pathological processes, including energy metabolism, stress response, cytoskeleton, synaptic transmission, signal transduction, and amino acid metabolism [15].

Network pharmacology, an approach capable of revealing the mechanisms of multiple drugs for diseases, makes the systematic study of herbal formulae achievable by interconnecting disease features, bioactive agents, and drug targets [16, 17]. Molecular docking is a computer simulation methods for predicting the binding affinity of a small molecule ligand and a protein. Since the advantages in analyzing the complex interaction between herbal components and the targets of disease [18, 19], network pharmacology and molecular docking-based strategy were applicable to investigate the multitarget mechanisms of SQYG on AD and help uncover drug action mechanisms.

In the present study, the active compounds of SQYG and common targets between SQYG and AD were screened from databases. The network pharmacology was applied to construct a herb-compound network and compound-target network and analyze the key nodes based on active compounds and targets. Enrichment analysis was performed to classify cellular components, biological processes, molecular functions, and KEGG pathways. Molecular docking was employed to analyze the binding affinity between main compounds and key targets. This study aimed to elucidate the pharmacological mechanisms of SQYG on AD.

## 2. Materials and Methods

### 2.1. Study Design

**Figure 1:**
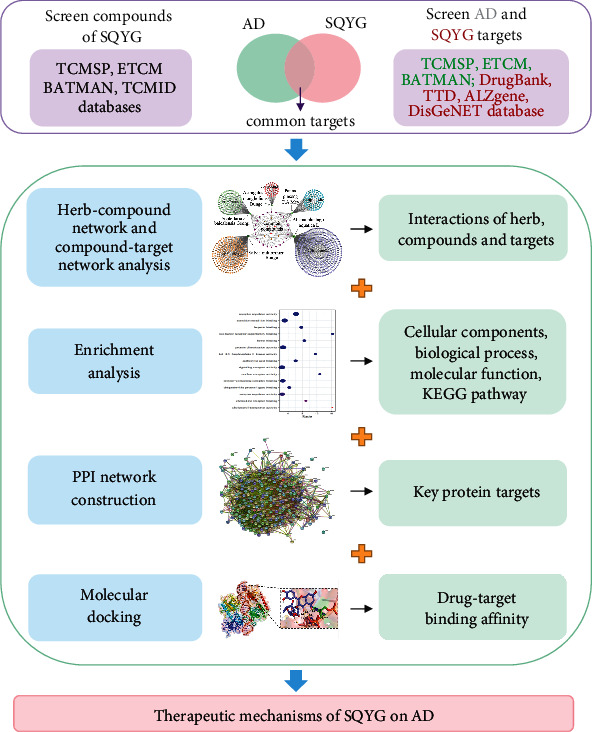
Schematic diagram of combining network pharmacology and molecular docking used in this study.

The active compounds of SQYG and common targets between SQYG and AD were screened from databases. The herb-compound network and compound-target network were constructed by the Cytoscape. The protein-protein interaction (PPI) network of common targets was constructed by the STRING. The Gene Ontology (GO) and KEGG enrichment analysis of common targets were performed by the SangerBox. The molecular docking was analyzed using the Autodock Vina and AutoDock. The schematic diagram of the study is shown in Figure 1.

### 2.2. Screening the Active Compounds of SQYG

The active compounds of SQYG were collected from multiple databases, including the Traditional Chinese Medicine Systems Pharmacology database (TCMSP, https://tcmspw.com/tcmsp.php), Traditional Chinese Medicine Integrated Database (TCMID, http://www.megabionet.org/tcmid/), The Encyclopedia of Traditional Chinese Medicine (ETCM, http://www.tcmip.cn/ETCM/index.php/Home/), and Bioinformatics Analysis Tool for Molecular MechANism of Traditional Chinese Medicine (BATMAN-TCM, http://bionet.ncpsb.org.cn/batman-tcm/) databases [18–21]. BATMAN-TCM was based on TCM ingredients' target prediction and subsequent network pharmacology analyses.

### 2.3. Screening the Targets of SQYG and AD

We hypothesize that the targets of SQYG intersect with the targets of AD were potential therapeutic targets of SQYG on AD. The protein targets retrieved from TCMSP databases were standardized using the UniProt Knowledgebase database (UniProt KB, http://www.uniprot.org/help/uniprotkb). The therapeutic targets for AD treatment were obtained from the DrugBank database (https://go.drugbank.com/), Therapeutic Target Database (TTD, http://db.idrblab.net/ttd/), ALZgene database (http://www.alzgene.org/), and DisGeNET database (https://www.disgenet.org/) [20, 21].

### 2.4. Herb-Compound Network and Compound-Target Network Construction

The herb-compound network helps to probe deeper into the interactions between herbs and corresponding compounds. The compound-target network was constructed for understanding closer correspondence between compounds and potential targets. The herb-compound network and compound-target network were constructed by the Cytoscape. The “degree” calculated by linked edges can well reflect the significance of nodes. The degree of each node and compounds were analyzed by the CytoHubba plugin in Cytoscape [22].

### 2.5. Gene Ontology (GO) and KEGG Enrichment Analysis

The enrichment analysis was performed by the SangerBox (http://sangerbox.com), which can classify cellular components, biological process, molecular function, and KEGG pathway. The results were used for post-analysis and visualization by the WebGestalt (http://www.webgestalt.org/).

### 2.6. Protein-Protein Interaction (PPI) Network Functional Enrichment Analysis

The STRING was applied to construct PPI networks using common targets between SQYG and AD [23]. Interactions with high confidence (interaction score >0.4) for PPI networks were employed for network visualization and analysis. The degree of each protein was calculated in the same manner. The protein subnetworks were constructed by the MultiContrast Delayed Enhancement (MCODE) [24].

### 2.7. Molecular Docking

The main compounds of SQYG and key protein targets were analyzed by molecular docking using the Autodock Vina and AutoDock [25]. The 3D structures of main compounds were obtained from the TCMSP database. The 3D structures of key protein targets were obtained from the RCSB Protein Data Bank (PDB) and AlphaFold (Table 1). The figures of the active binding site were generated with the PyMOL software [26]. Heatmaps were graphed according to the binding affinity between active compounds and key targets using Graph Pad Prism 5.0.

## 3. Results

### 3.1. Screened Active Compounds of SQYG

**Figure 2:**
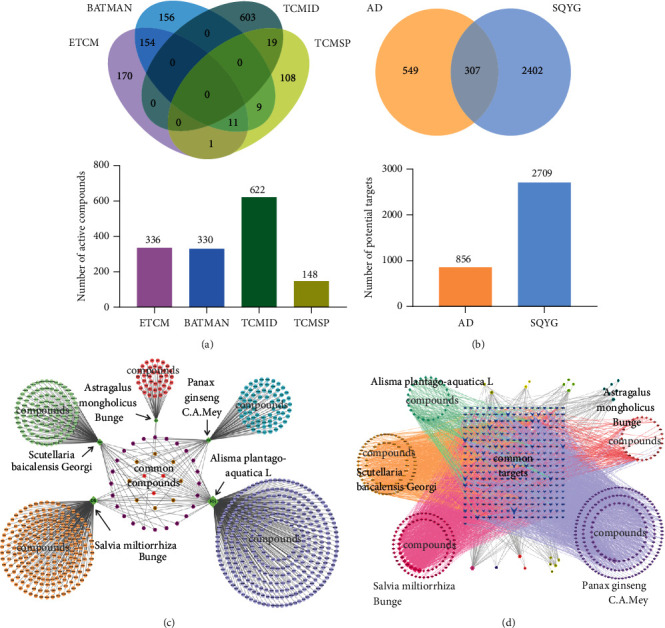
Potential compounds, targets prediction and network construction. (a) Number of screened compounds from 4 databases were indicated as Venn diagrams. (b) Venn diagram of predicted targets of AD and SQYG. (c) Herb-compound network of SQYG. (d) Compound-target network of SQYG.

A total of 1436 compounds of SQYG were collected, including 365 compounds in ETCM, 330 compounds in BATMAN, 622 compounds in TCMID, and 148 compounds in TCMSP databases (Figure 2(a)). After removing duplicates, 816 compounds were collected, including 365 compounds in *Panax ginseng* C.A.Mey, 95 compounds in *Astragalus mongholicus* Bunge, 126 compounds in *Scutellaria baicalensis* Georgi, 237 compounds in *Salvia miltiorrhiza* Bunge, and 42 compounds in *Alisma plantago-aquatica* L (Table 2). The screened active compounds of each herb were listed and are ranked by degree in Supplementary Table 1.

### 3.2. Screened Common Targets between SQYG and AD

In order to screen the potential targets, the screened 816 compounds of SQYG were uploaded to TCMSP, BATMAN, and ETCM databases. 895 and 1727 target genes were collected from BATMAN and ETCM databases, respectively. 282 protein targets were collected from TCMSP database. After removing duplicates, a total of 2709 targets were obtained. A total of 856 AD-related targets were screened by retrieving DrugBank, TTD, ALZGene, and DisGeNET databases with a relevance score ≥0.3. There were 681, 66, 141, and 123 protein targets obtained from ALZGene, DrugBank, TTD, and DisGeNET databases, respectively. Overlap between targets of SQYG and AD, 307 targets of SQYG were found to be AD-related targets and presented by Venn diagrams (Figure 2(b)). These 307 common targets could be potential therapeutic targets for SQYG in AD treatment. Further bioinformatic analysis of these common targets were helpful in revealing the therapeutic mechanisms of SQYG on AD.

### 3.3. Herb-Compound Network and Compound-Target Network Analysis

To demonstrate the relationships between compounds and each herb, a herb-compound network was constructed. The common compounds in central circular area were presented in multiple herbs (Figure 2(c)). The active compounds and common targets were used to construct the compound-target network and the common targets were marked with blue in a center square region (Figure 2(d)). In both networks, herbs, compounds and targets were represented by nodes and linked with the edges.

### 3.4. Enrichment Analysis of Common Targets

#### 3.4.1. Cellular Components Analysis

**Figure 3:**
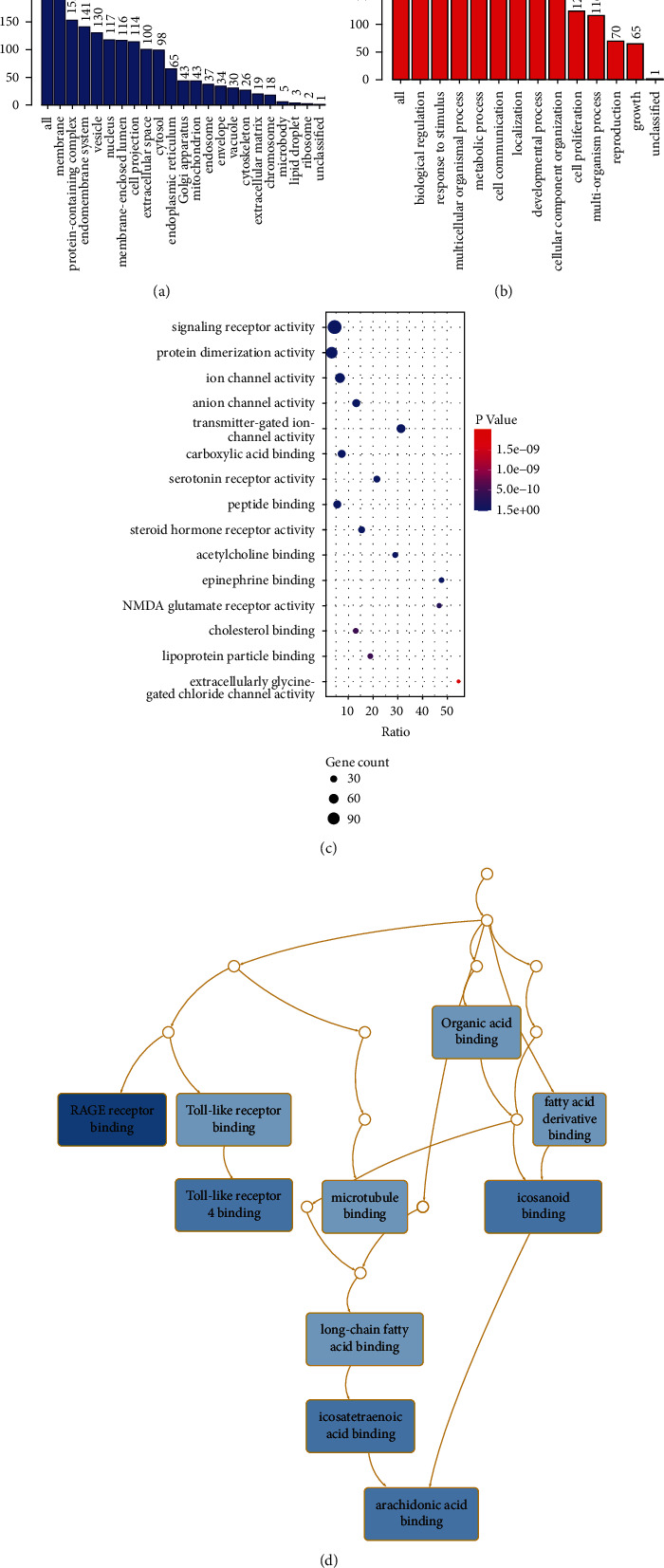
Enrichment analysis results of common targets. The GO enrichment results of cellular component (a) and biological process (b). (c) Bubble map of molecular function analysis. (d) Directed acyclic graph (DAG) of the KEGG pathway. The darker the blue, the higher the degree of enrichment.

There were 42 GO terms enriched in cellular components. The main cellular components included integral components of the plasma membrane, synapse part, dendritic tree, neuronal cell body, transporter complex, membrane region, an intrinsic component of postsynaptic membrane, an intrinsic component of the presynaptic membrane, GABA receptor complex, and integral component of postsynaptic specialization membrane (Figure 3(a)).

#### 3.4.2. Biological Process Analysis

There were 93 GO terms were enriched in biological processes. The main biological process included circulatory system process, lipid localization, response to the metal ion, response to molecule of bacterial origin, hormone transport, response to the antibiotic, steroid metabolic process, G protein-coupled receptor signaling pathway, coupled to cyclic nucleotide second messenger, and ammonium transport (Figure 3(b)).

#### 3.4.3. Molecular Function Analysis

There were 68 GO terms enriched in molecular functions. The bubble map provides a graphical representation of the highly enriched terms of molecular function (Figure 3(c)). The main molecular function included signaling receptor activity, protein dimerization activity, substrate-specific channel activity, chloride channel activity, transmitter-gated ion channel activity, carboxylic acid-binding, serotonin receptor activity, peptide binding, steroid hormone receptor activity, and acetylcholine binding. The involved proteins of each molecular function are listed in Supplementary Table 2.

#### 3.4.4. KEGG Pathway Analysis

There were 119 pathways obtained from KEGG enrichment analysis. The top 10 enriched pathways are shown in Figure 3(d) and Supplementary Table 3, including RAGE receptor binding, arachidonic acid-binding, Toll-like receptor 4 binding, icosanoid binding, icosatetraenoic acid binding, Toll-like receptor binding, long-chain fatty acid binding, organic acid-binding, microtubule binding, and fatty acid derivative binding.

### 3.5. PPI Network Analysis of Common Targets

**Figure 4:**
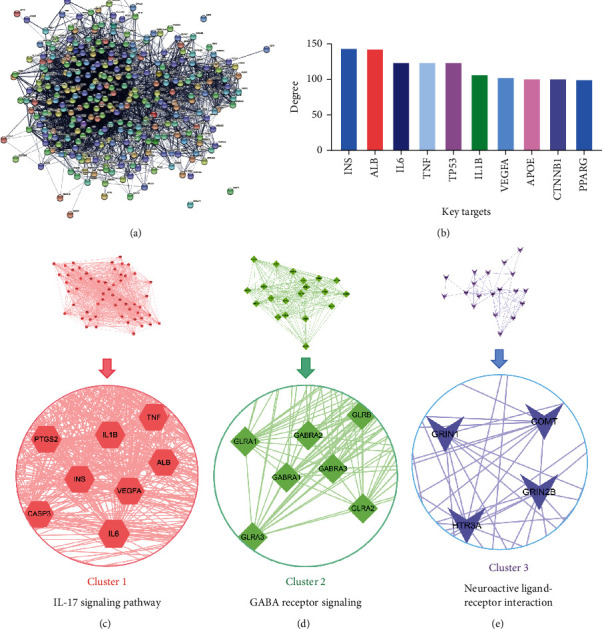
PPI network construction and subnetwork analysis. (a) PPI network of common protein targets of SQYG. (b) Bar plot of the key protein targets. The *y*-axis represents the number of neighboring proteins of the protein target. The *x*-axis represents the protein target. (c) Cluster 1 and its core protein targets. (d) Cluster 2 and its core protein targets. (e) Cluster 3 and its core protein targets.

The PPI network consists of 307 nodes and 4780 edges, with an average node degree of 31.1 (Figure 4(a)). The key protein targets with high degree are shown in Figure 4(b)). The PPI network contained 3 clusters, including module 1 (MCODE score = 43.382) (Figure 4(c)), module 2 (MCODE score = 20) (Figure 4(d)), and module 3 (MCODE score = 6.67) (Figure 4(e)). The details of each cluster are shown in Table 3. The clusters 1, 2, and 3 were related to the IL-17 signaling pathway, GABA receptor signaling, neuroactive ligand-receptor interaction, respectively.

### 3.6. Molecular Docking Analysis

**Figure 5:**
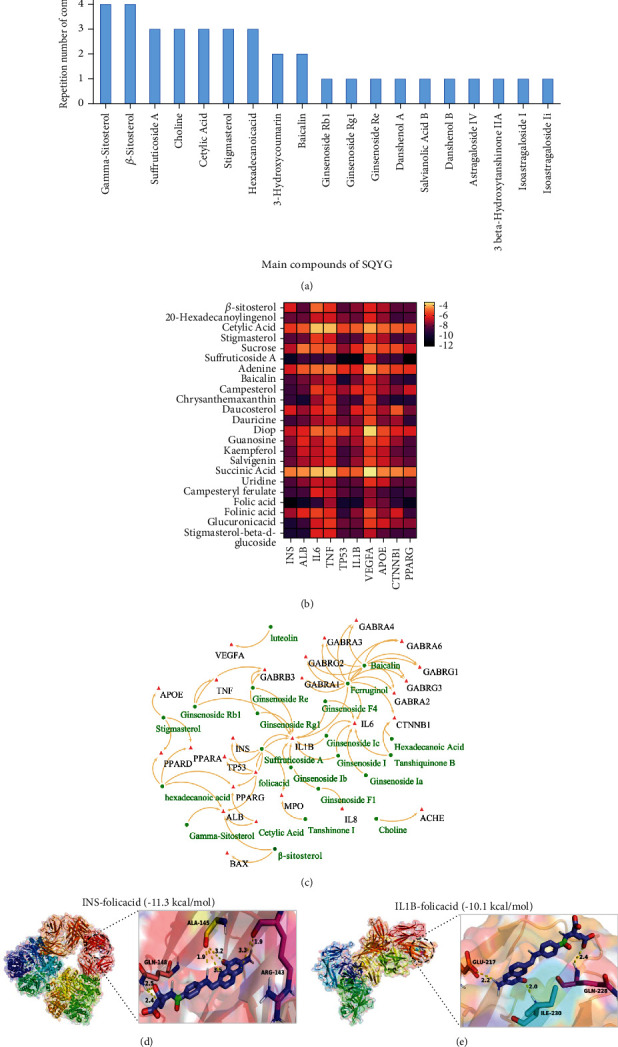
Main compounds of SQYG and drug-target interaction prediction. (a) Repetition number of main compounds of five herbs in SQYG. (b) Heat map of molecular docking scores based on drug-target binding affinity. (c) Drug-target interaction prediction between main compounds and key protein targets. The green nodes represented main compounds of SQYG and the red nodes represented the key protein targets. (d-e) Representative schematic diagrams of drug-target molecular docking.

**Figure 6:**
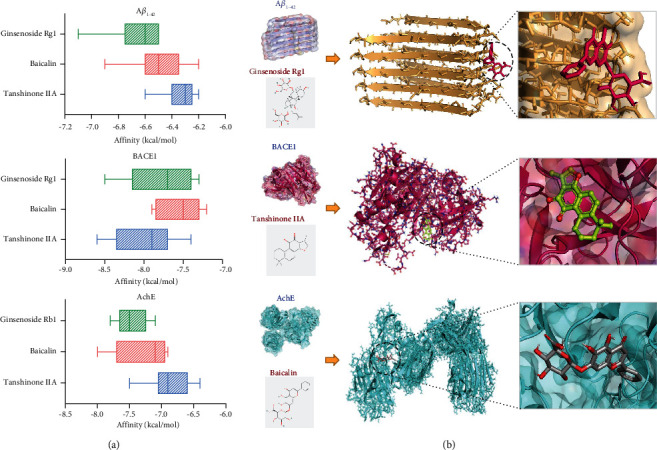
Molecular docking results between 3 representative compounds (Ginsenoside Rg1, Baicalin, Tanshinone IIA) of SQYG and 3 representative AD protein targets (A*β*_1–42_, BACE1, AchE). (a) Binding affinity of compound-target molecular docking. (b) Representative schematic diagram of compound-target molecular docking.

The main compounds of SQYG are shown in Figure 5(a). These compounds and key targets were selected for molecular docking analysis. The drug-target binding affinity and interaction between main compounds and key targets were indicated in heat map and interaction network, respectively (Figure 5(b)-5(c)). The molecular docking results of INS-folicacid and IL1B-folicacid are shown in Figures 5(d)-5(e) as representative results. A*β*_1–42_, BACE1, and AchE were commonly recognized as the classical therapeutic targets in AD. The drug-target binding affinity and the best-scored docked position between these therapeutic targets and 3 representative compounds (ginsenoside Rg1, baicalin, and tanshinone IIA) are indicated in Figures 6(a)-6(b).

## 4. Discussion

TCM has been used in clinical practice for several thousand years. TCM-based new drug development for treating complex diseases is promising. However, it is difficult to reveal molecular mechanisms of the Chinese herbal prescription due to the diverse ingredients and their complex interaction with the human body [27]. Modern pharmacological research methods including network pharmacology, bioinformatics, and molecular docking contributed to understanding the “multicomponent, multitarget, and multipathway” of TCM, and provided valuable clues for subsequent experimental validation. SQYG has been applied to ameliorate cognitive impairment in mild-to-moderate AD patients. However, the underlying therapeutic mechanisms remain incompletely understood.

In this study, network pharmacology, bioinformatics, and molecular docking network were applied to investigate the underlying pharmacological mechanisms of SQYG on AD. The main compounds of SQYG included gamma-sitosterol, *β*-sitosterol, suffruticoside A, choline, cetylic acid, stigmasterol, hexadecanoicacid, 3-hydroxycoumarin, baicalin, ginsenoside Rb1, ginsenoside Rg1, ginsenoside Re, danshenol A, salvianolic acid B, danshenol B, astragaloside IV, 3 beta-hydroxytanshinone IIA, and isoastragaloside I. The PPI network results indicated that INS, ALB, IL6, TNF, TP53, IL1B, VEGFA, APOE, CTNNB1, and PPARG were key targets of SQYG on AD. The compounds (ginsenoside Rg1, baicalin, and tanshinone IIA) were detected by high-performance liquid chromatography (HPLC) in previous research. They were also screened by network pharmacology from TCM database. So, they were selected for further molecular docking with classical AD targets.

GO enrichment analysis was used to explore the distribution of the overall proteins and grasp the correlation between proteins and biological function in a whole [28–30]. In this study, GO enrichment analysis was applied to classify cellular components, biological process, molecular function, and KEGG pathway of the common protein targets between SQYG and AD. The results indicated that the common protein targets have multiple biological functions, including lipid localization, response to the metal ion of biological process, synapse part, neuronal cell body, GABA receptor complex of biological process, transmitter-gated ion channel activity, peptide binding, and acetylcholine binding of molecular function.

**Figure 7:**
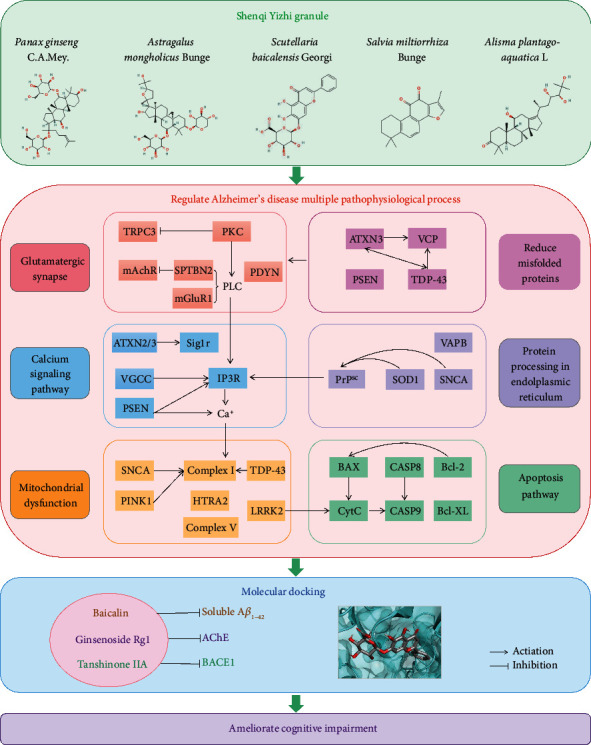
Putative schematic model of pharmacological mechanisms of SQYG ameliorating cognitive impairment of AD. The predominant protein targets of SQYG were related to regulating multiple AD pathophysiological processes, principally involving glutamatergic synapse, calcium signaling pathway, mitochondrial dysfunction, reduction of misfolded proteins, protein processing in endoplasmic reticulum, and apoptosis pathway. Molecular docking focused on the 3 representative compounds and 3 prime targets in AD pathogenesis. These active therapeutic targets and involved biological processes of SQYG ultimately improve cognitive function.

There were a lot of metabolic, regulatory, and signal transduction pathways in the organism. KEGG pathway enrichment analysis allowed us to identify important biochemical-metabolic pathways and signal transduction pathways in which the key proteins were involved [31]. In this study, the IL-17 signaling pathway, GABA receptor signaling, neuroactive ligand-receptor interaction, RAGE receptor binding, Toll-like receptor binding, and long-chain fatty acid binding were the main pathways where SQYG regulates for therapeutic effects. These key proteins, biological functions, and signaling pathways collectively revealed the therapeutic mechanisms of SQYG on AD. The putative schematic model of pharmacological mechanisms of SQYG ameliorating cognitive impairment of AD is shown in Figure 7. The key targets and compounds are discussed in detail below.

The transcription factor TP53, commonly known as p53, can initiate apoptosis. Hypoxia, DNA damage, oncogene activation, microtubule destruction, and oxidative damage all caused an increase in the p53 expression [32]. Overexpression of p53 caused apoptosis in cultured rat hippocampal pyramidal neurons [33]. TP53 induced neuronal apoptosis via transcription-associated molecular pathways that regulate apoptosis-dependent targets [34]. The mitophagy were downregulated by TP53 [35]. It is worth noting that p53 expression levels in cerebral gray matter were positive correlated with A*β* in PS/APP mice [36]. Corresponding to that, dexmedetomidine was against A*β* by suppressing the TP53 expression [37].

Interleukin-1 beta (IL1B) is ubiquitously distributed in the hippocampus and hypothalamus [38]. IL1Bs were mainly released in the brain by microglia and astrocytes, which have critical roles in the immune response. These signaling molecules mediated neuronal proliferation, differentiation, and apoptosis and induced late long-term potentiation [39]. Neuroinflammation plays a critical role in the pathophysiology of AD and is partly characterized by increased pro-inflammatory cytokines such as IL1B [40]. Moreover, IL1B levels were elevated in the brain and serum of patients with AD [41].

The peroxisome proliferator-activated receptor-*γ* (PPAR*γ*) is a member of the PPAR family. The activation of PPAR*γ* controlled the multiple pathways involved in inflammation and lipid metabolism. In addition, the vital role of PPAR*γ* agonists in neuroprotection has been extensively studied in neurodegeneration, such as in A*β*-induced AD. Also, in the light of the present evidence, the neuroprotective effects shown by agonists of PPAR*γ* were related to the capability of decreasing A*β* levels [42]. Overall, PPAR*γ* has a wide spectrum of functions in nerve inflammation [43], energy metabolism, cerebrovascular protection [44], and reducing oxidative stress [45], which may significantly improve AD-induced cognitive impairment.

Baicalin has been shown to have anti-inflammation and neuroprotective effects. Baicalin effectively improved A*β*-induced learning and memory deficit, hippocampus injury, and neuron apoptosis. The neuroprotective mechanisms were related to preventing the decrease of A*β*-induced mitochondrial membrane potential, cytochrome c release, and caspase-9/-3 activation [46]. Besides, baicalin alleviated microglia-induced neuroinflammation by inhibiting NLRP3 inflammasome activation and the TLR4/NFB signaling pathway. Baicalin has been shown to effectively reduce the number of activated microglia and pro-inflammatory cytokines in A*β*‐stimulated BV2 microglial cells [47].

Folic acid deficiency is linked to cognitive deterioration and AD [48]. The folic acid levels in serum and cerebrospinal fluid of AD patients were decreased [49–52]. Randomized clinical trials have shown that folate supplementation can enhance cognition and social function in older people with neuropsychiatric disorders [52, 53]. The APP, PS1, and A*β* protein levels in APP/PS1 mice hippocampus were found to be elevated due to the lack of folate [54]. Moreover, folic acid could reduce the deposition of A*β*_42_ by decreasing the mRNA and protein expressions of *β*-secretase and *γ*-secretase complex catalytic component in the APP/PS1 mice brain [55].

Dauricine is a bisbenzylisoquinoline alkaloid derivative extracted from the rootstock of *Menispermum dauricum* DC. Dauricine alleviated cognitive deficits in 3xTg-AD mice by lowering A*β* plaques and hyperphosphorylated tau and raising hippocampus ATP levels. After dauricine administration, Aco2, Ndufs1, Cox5a, and SDHB involved in the mitochondrial energy metabolism were significantly increased, while the expression level of synapse-related proteins such as Syn1 and Syn2 were upregulated [56]. Dauricine regulated the proteins levels of Nrf2 and Kelch-like ECH-associated protein 1 (Keap1) that is necessary for the activation of Nrf2 in APPsw cells [57]. Overall, dauricine is beneficial to inhibit inflammatory reaction and apoptosis.

However, there were still limitations in the present study. The screened active compounds of SQYG lack analytical chemistry identification. Moreover, the key targets and potential pathways screened by enrichment analysis require experimental validation.

## 5. Conclusion

In conclusion, the present study screened 816 active compounds and 307 common targets of SQYG on AD and constructed compound-target network and PPI network using network pharmacology analysis. SQYG alleviated cognitive impairment of AD by regulating lipid metabolism, metal ion metabolism, IL-17 signaling pathway, GABA receptor signaling, and neuroactive ligand-receptor interaction. The molecular mechanisms of SQYG improving the cognitive function were associated with high binding affinity between ginsenoside Rg1 and A*β*_1–42_, tanshinone IIA and BACE1, and baicalin and AchE. This study provides insights into pharmacological mechanisms of SQYG in ameliorating the cognitive impairment of AD.

## Figures and Tables

**Table 1:** Candidate targets for molecular docking.

Gene symbol	Description	PDB ID	AlphaFold
INS	Insulin	6B70	
ALB	Albumin	1YSX	
IL6	Interleukin-6		P05231
TNF	Tumor necrosis factor		P01375
TP53	Cellular tumor antigen p53	3Q05	
IL1B	Interleukin-1 beta	4DEP	
VEGFA	Vascular endothelial growth factor-A		P15692
APOE	Apolipoprotein E	1BZ4	
PPARG	Peroxisome proliferator-activated receptor gamma	1JPW	
CTNNB1	Catenin beta-1	3E00	

**Table 2:** Number of compounds from five herbs of SQYG after removing duplicates.

Herbs	BATMAN	ETCM	TCMID	TCMSP	Total
*Panax ginseng* C. A. Mey	155	157	293	22	365
*Astragalus membranaceus* (fisch.) bunge	35	27	70	20	95
Scutellaria baicalensis georgi	65	54	92	36	126
Salvia miltiorrhiza bunge	75	96	166	65	237
Alisma plantago-aquatica linn	21	31	33	10	42
Total	330	336	622	148	816

**Table 3:** Clustering analysis results of PPI network. 3 subnetworks were identified, and the details of each subnetwork were listed.

Cluster	Targets	Count	Score
1	AGTR1, AGT, GSK3B, SIRT1, MPO, CCL2, APOE, CRP, IL1B, ICAM1, APP, PPARG, ALB, NOS3, SOD2, LEP, CAV1, MMP9, IL10, IL6, INS, TNF, IL4, PPARA, MAPK14, NR3C1, TP53, CASP3, NFE2L2, HSP90AA1, CTNNB1, NOS2, PTGS2, CDKN2A, IKBKB, RELA, ESR1, NFKBIA, HIF1A, VEGFA, CXCL10, ACE, CCL5, HMOX1, CASP8, TGFB1, SERPINE1, IFNG, CAT, REN, SMAD3, MMP1, FOS, MMP3, IGF1, PLG	56	43.382

2	GABRB2, GABRB1, GABRB3, GABRA6, GLRB, GABRA2, GABRQ, GLRA3, GABRA3, GABRA4, GABRE, GABRG1, GLRA1, GABRG2, GABRP, GABRA1, GABRD, GABRG3, GLRA2, GABRA5	20	20

3	SLC6A4, GRIN2D, GRIN2C, GSTM1, GRIN1, COMT, DRD4, CHRM3, CHRM2, HTR3A, GSTP1, CHRM1, CHRM5, MAOB, GRIN3B, TPH1, GRIN3A, GRIN2B, GRIA1	19	6.667

## Data Availability

The data of this study are accessible upon an appropriate request from the corresponding author.

## Supplementary Materials

Supplementary Materials Table S1: The screened active compounds of five herbs of SQYG. Table S2: Top 10 terms of molecular function of common targets. Table S3: Top 10 terms of KEGG pathway of common targets.
