# Supplementary material for: Network Pharmacology and Molecular Docking-Based Strategy to Investigate the Multitarget Mechanisms of Shenqi Yizhi Granule on Alzheimer's Disease
Source: Evid Based Complement Alternat Med. 2022 Apr 30;2022:8032036. doi: 10.1155/2022/8032036 (PMC9078761; doi:10.1155/2022/8032036)
Supplement: Supplementary Materials — Table S1: The screened active compounds of five herbs of SQYG. Table S2: Top 10 terms of molecular function of common targets. Table S3: Top 10 terms of KEGG pathway of common targets. [file 8032036.f1.docx]

# Supplementary Materials

**Supplementary Table 1**: The screened active compounds of five herbs of SQYG.

| **Compounds** | **Degree** |
| --- | --- |
| ***1. Panax ginseng* C.A.Mey** |  |
| Gamma-Sitosterol, Sitosterol,Î’-Sitosterol, β-sitosterol. | 4 |
| 20-Hexadecanoylingenol, Cetylic Acid, Hexadecanoic Acid, Palmitic Acid, Choline, Hexadecanoicacid, Stigmasterol, Sucrose, Suffruticoside A. | 3 |
| 3-Hydroxycoumarin,Folic Acid, 3-o-beta-d-glucuronopyranosyl gypsogenin, Adenine, Adeninenucleoside, Alexandrin, Daucosterol, Caproic Acid, Eleutheroside A, Sitogluside, S trumaroside, Î’-Sitosterol-Î’-D-Glucoside, beta-sitosterol, Campesterol, Campesterol, M-Cresol, campesteryl ferulate, Chrysanthemaxanthin, Daucosterol, Dauricine, Diop, Folicacid, Folinic acid, Glucuronic Acid, Glucuronicacid, Kaempferol, Stigmasterol-beta-d-glucoside, Succinic Acid. | 2 |
| Maalioxide, (1Ar,7R,7Ar,7Bs)-1,1,7,7A-Tetramethyl-1A,2,3,5,6,7,7A,7B-Octahydro-1H-Cyclopropa[A]Naphthalene,Calarene, (1R,9S)-4,11,11-Trimethyl-8- Methylenebicyclo[7.2.0]Undec-4-Ene,Caryophellene,Î’-Caryophyllene, (1S,2S)-2-Isopropenyl-4-Isopropylidene-1-Methyl-1-Vinylcyclohexane,Î“-Elemene, (9R,10S)-Epoxyheptadecan-4,6-Diyn-3-One, (S)-1-Methyl-4-(6-Methylhepta-1,5-Dien-2-Yl)Cyclohex-1-Ene,Î’-Bisabolene, 12-O-Nicotinoylisolineolone, 16-Oxoseratenediol, 1-Heptadecanol, 1-Methoxy-(9R,10S)-Epoxyheptadecan-4,6-Diyn-3-One, 1-Tetradecanol, 2,5-Dimethyl-7-Hydroxy Chromone, 2,6-Ditertbutyl-4-Methyl Phenol, 20(R)-Ginsenoside Rg3, 20(R)-Ginsenoside Rh1, 20-(r)-Ginsenoside-rg2, 20(r)-Ginsenosiderh1, 20(r)-Ginsenoside-rh1, 20(R)-Ginsenoside-Rh2, 20(S)-Ginsenoside Rg3, 20(s)-Ginsenosiderg3, 20-(s)-Ginsenoside-rg3, 20(s)-Ginsenosiderh1, 20(s)-ginsenoside-rh1, 20(s)-ginsenosiderh2, 20(S)-Protopanaxadiol, 20-Glucosylginsenoside Rf, 20-glucosylginsenosiderf, 2-Heptadecanone, 2-Methyl-tetradecane, 3,4-Dihydroxybenzaldehyde,Hydroxybenzoic Acid, M-Hydroxybenzoic Acid,P-Hydroxybenzoic Acid,Salicylic Acid, 3,5-Dimethyl-4-Methoxybenzoic Acid, 5-(heptadec-12-enyl)Resorcinol, 6'-Malonylginsenoside Rd1, 6-Malonylginsenoside rd1, 6''-Malonylginsenosiderd1, 7-(beta-xylosyl)Cephalomannine, 7alpha-l-rhamnosyl-6-methoxylutcolin, Adenosine, Adenosine Triphosphate, Adenosine,Adenine Nucleoside, Alexandrin_qt, Alloaromadendrene, Alpha-Cadinol, Alpha-Farnesene, Alpha-Guriunene, Alpha-guttiferin, Alpha-Humulene,Humulene,Î‘-Humulene, Aposcopolamine, Aposiopolamine, Arachidonate, Araloside A, Argininyl-Fructosyl-Glucose, Bata-Caryophyllene, Beta-Bisabolene, Beta-Elemene, Beta-Elemene,Î’-Elemene, Beta-Humulene, Beta-Santalol, Beta-Selinene, Bicyclogermacrene, Biotin, Calarene, Cedrol,Eudesmol,Î‘-Cedrol, Celabenzine, Chikusetsusaponin Iii, Chikusetsusaponin Iv, Chloropanaxydiol, Cis-9,Cis-12-Linoleic Acid, Inositol, Linoleic, Linoleic Acid, Cis-9,cis-12-linoleicacid, Cis-widdrol alpha-epoxide, Citric Acid, Citronellal, Delta-Elemene, Delta-Guaiene, Deoxygomisin A, Deoxyharringtonine, Dianthoside, Dianthramine, Dibutyl Oxalate, Dibutyl Phthalate, diterbutyl phthalate, D-Mannuronic Acid, Dodecane, Dodecanol, Eicosane, eicosanetetraenoic acid, Elemicin, Epsilon-Cadinene, Fructose, Frutinone A, Fumarine, Gamma-Selinene, Ginsenol, Ginsenoside F1, Ginsenoside F4, Ginsenoside I, Ginsenoside Ia, Ginsenoside Ib, Ginsenoside Ic, Ginsenoside Ii, Ginsenoside Iii, Ginsenoside La, Ginsenoside R0, Ginsenoside Ra0, Ginsenoside Ra1, Ginsenoside Ra2, Ginsenoside Ra3, Ginsenoside rb0, Ginsenoside Rb1, Ginsenoside Rb2, Ginsenoside Rb3, Ginsenoside Rc, Ginsenoside Rd, Ginsenoside Re, Ginsenoside Rf, Ginsenoside Rg1, Ginsenoside Rg2, Ginsenoside Rg3, Ginsenoside Rg5_qt, Ginsenoside Rh2, Ginsenoside Rh3, Ginsenoside Rh4, Ginsenoside Rs1, Ginsenoside Rs2, Ginsenoside-La, Ginsenoside-r0, Ginsenosidera0, Ginsenoside-ra0, Ginsenosidera1, ginsenoside-ra1, Ginsenosidera2, Ginsenoside-ra2, Ginsenosidera3, Ginsenoside-ra3, Ginsenosiderb1, Ginsenoside-Rb1, Ginsenosiderb2, Ginsenoside-Rb2, Ginsenosiderb3, Ginsenoside-rb3, Ginsenosiderc, Ginsenoside-Rc, Ginsenosiderd, Ginsenoside-Rd, Ginsenosidere, Ginsenoside-Re, Ginsenosiderf, Ginsenoside-rf, Ginsenosiderg1, Ginsenoside-rg1, Ginsenosiderg2, Ginsenoside-Rh1, Ginsenosiderh3, Ginsenoside-rh3, Ginsenosiderh4, Ginsenoside-rh4, Ginsenoside-Rh4_qt, Ginsenosidero, Ginsenosiders1, Ginsenoside-rs1, Ginsenosiders2, Ginsenoside-rs2, Ginsenoyne A, Ginsenoyne B, Ginsenoyne C, Ginsenoyne D, Ginsenoyne E, Ginsinsene, Girinimbin, Girinimbine, Gomisin A, Gomisin B, Heptadecane, Hexadecane, Hexadecanoic Acid, Humulene, Humulene Epoxide I, Î‘-Cadinene, Î‘-Guaiene, Î‘-Guriunene, Î‘-Muurolene, Î‘-Neoclovene, Î‘-Panasinsene, Î‘-Santalol, Î‘-Selinene, Î’-Farnesene, Î’-Humulene, Î’-Maaliene, Î’-Neoclovene, Î’-Panasinsene, Î’-Patchoulene, Î’-Selinene, Î’-Sitosterol-3-(6-Linoleoyl)Glucopyranoside, Î’-Sitosterol-3-(6-Palmitoleoyl)Glucopyranoside, Î’-Sitosterol-3-(6-Stearoyl)Glucopyranoside, Î“-Cadinene, Î“-Selinene, Î”-Cadinol,Î”-Cadinene, Î”-Elemene, Inermin, Isocitric Acid, Isocitric Acid B, Isocitric Acid C, Isocitric Acid D, Isocitricacid, Isocitricacid b, Isocitricacid c, Isocitricacid d, Isocnidilide, kaempferol-3-arabofuranoside, Malic Acid, Malicacid, Malkangunin, Malonylginsenoside Rb2, Malonylginsenoside Rc, Malonylginsenoside Rd, malonylginsenosiderb2, Malonylginsenosiderc, malonylginsenosiderd, Maltose, Maltose-b, Malvic Acid, Mannose, Mannose-B, Menthyl Acetate, Methyl Palmitate, Mycosinol, Neointermedeol, Niacin,Nicotinic Acid, Nicotinicacid, N-Octane, Nonacosane, Nonacosanediol-6,8, Nonadecane, Notoginsenoside R1, Notoginsenoside R2, Notoginsenoside R4, Notoginsenoside r6, N-Pentadecane, N-Tridecane, Octanal, Palmitic Acid, Palmitin, Palmitoleic Acid, Panacon, Panaginsene, Panasinsanol A, Panasinsanol B, Panaxacol, Panaxadiol, Panaxatriol, Panaxene, Panaxydol, Panaxynol, Panaxytriol, Pancratistatin, Pandamine, Patchouli Alcohol, Pentadecane, Pentadecanoic Acid, P-Glucosyloxymandelonitrile, Protopanaxadiol, Protopanaxatriol, Protopanaxtriol, Protopine, Pseudoginsenoside F11, Pseudohypericin, Putrescine, Pyrrole-2-Aldehyde, Pyruvic Acid, pyruvicacid, Quinquenoside R1, Quinquenosider1, Raffinose, Ramalic Acid, Rhamnose, Riboflavine, Salicylic acid, Selina-4(15),7(11)-Diene, Se-Methyl-L-Selenocysteine, Spermidine, Spermine, Stigmast-3-O-Î’-D-Glucopyanosyl-6-Hexadecanoate, Stigmast-3-o-β-d-glucopyanosyl-6-hexa-decanoate, Stigmasterol-3-(6-Linoleoyl)Glucopyranoside, Stigmasterol-3-(6-Oleoyl)Glucopyranoside, Stigmasterol-3-(6-Stearoyl)Glucopyranoside, Succinicacid, Suchilactone, Tartaric Acid, tartaricacid, Tauremisin, Tetradecane, trans-9-trans-12-linoleic acid, Trans-Caryophyllene, Tridecanoic Acid, Trifolin, Trifolirhizin, Vitamin B1, Vitamin B12, Vitamin B15, Vitamin B5, Widdrol, Xylose, α-cadinene, α-cedrol, α-guaiene, α-guriunene, α-humulene, α-muurolene, α-neoclovene, α-panasinsene, α-santalol, α-selinene, β-bisabolene, β-caryophyllene, β-elemene, β-farnesene, β-humulene, β-maaliene, β-neoclovene, β-panasinsene, β-patchoulene, β-selinene, β-sitosterol-3-(6-linoleoyl)glucopyranoside, β-sitosterol-3-(6-palmito leoyl)glucopyranoside, β-sitosterol-3-(6-stearoyl) glucopyranoside, γ-cadinene, γ-elemene, γ-selinene, δ-cadinene, δ-elemene. | 1 |
| ***2. Astragalus membranaceus* (Fisch.) Bunge** |  |
| Gamma-Sitosterol, Sitosterol,Î’-Sitosterol, β-sitosterol. | 4 |
| 20-Hexadecanoylingenol, Cetylic Acid, Hexadecanoic Acid, Palmitic Acid, Choline, Hexadecanoicacid, Sucrose, Suffruticoside A. | 3 |
| 3-Hydroxycoumarin, Folic Acid, 3-o-beta-d-glucuronopyranosyl gypsogenin, Adenine, adeninenucleoside, Beta-Sitosterol, Chrysanthemaxanthin, Folicacid, Folinic acid, Glucuronic Acid, Glucuronicacid, Kaempferol. | 2 |
| (?)-medicarpin, (3r)-2', 3'-dihydroxy-7, 4'-dimethoxyisoflavone, (3R)-3-(2-hydroxy-3,4-dimethoxyphenyl)chroman-7-ol, (3S,8S,9S,10R,13R,14S,17R)-10,13- dimethyl-17-[(2R,5S)-5-propan-2-yloctan-2-yl]-2,3,4,7,8,9,11,12,14,15,16,17-dodecahydro-1H-cyclopenta[a]phenanthren-3-ol, (6ar, 11ar)-10-hydroxy-3, 9-dimethoxypterocarpane, (6aR,11aR)-9,10-dimethoxy-6a,11a-dihydro-6H-benzofurano[3,2-c]chromen-3-ol, 1,7-Dihydroxy-3,9-dimethoxy pterocarpene, 2', 4'-dihydroxy-5, 20(r)-21,24-cyclo-3beta,25-dihydroxyl-dammar-23(24)-en-21-one, 2'-hydroxy-3, 2'-hydroxy-3',4'-dime thoxy-isoflavane-7-o-β-d-glucoside, 2'-Hydroxy-3',4'-Dimethoxy-Isoflavane-7-O-Î’-D-Glucoside, 2-Hydroxy-3-Methoxystrychnine, 3,5-Dimethoxystilbene, 3,9-di-O-methylnissolin, 3'-hydroxy-4--methoxyisoflavone-7-o-beta-d-glucoside, 4'-dimethoxyisoflavane-7-o-beta-d-glucoside, 4-hydroxy-2,6-dimethyl-6-(3,7-dimethyl-2,6-octadienyl)-8- (3-methyl-2-butenyl)-2h-1-benzopyran-5,7(3h,6h)-dione, 4-Hydroxycoumarin,Folinic Acid, 5'-hydroxyiso-muronulatol-2',5'-di-O-glucoside, 6-dimethoxy-isoflavane, 7-O-methylisomucronulatol, 9, 10-dimethoxypterocarpane-3-o-beta-d-glucoside, 9,10-dimethoxypterocarpan-3-O-β-D-glucoside, 9,10-Dimethoxy-Pterocarpane-3-O-Î’-D-Glucoside, 9,10-dimethoxy-pterocarpane-3-o-β-d-gluco-side, Acetic Acid, Acetyl Astragaloside I, acetylastragaloside, astragaloside 1~8, Astragaloside I, Astragaloside Ii, Astragaloside Iii, Astragaloside Iv, Astragaloside V, Astragaloside Vi, Astragaloside Vii, Astragaloside Viii, astramembrannin, Astramembrannin I, Astramembrannin Ii, Astrasieversianin Ix, Betaine, Bifendate, Calycosin, Canavanine, Cycloastragenol, Cyclosieversigenin, FA, Folinicacid, Foliosidine, Formononentin, Formononetin, hederagenin, Î’etaine, Isoastragaloside I, Isoastragaloside Ii, Isoastragaloside, 1,3,isoflavanone, Isomucronulatol-7,2'-di-O-glucosiole, Isorhamnetin, Jaranol, Kumatakenin, Kumugansine a, Lupeol, Mairin, Medicarpin, N-candicine, Quercetin, Rhamnocitrin, Soyasapogenol B, Soyasaponin 1, β-etaine. | 1 |
| ***3. Scutellaria baicalensis* Georgi** |  |
| Gamma-Sitosterol, Sitosterol,Î’-Sitosterol, β-sitosterol. | 4 |
| 20-Hexadecanoylingenol, Cetylic Acid, Hexadecanoic Acid, Palmitic Acid, Hexadecanoicacid, Stigmasterol, Sucrose, Suffruticoside A. | 3 |
| Baicalin, Beta-sitosterol, Campesterol, Campesterol, M-Cresol, Campesteryl ferulate, Diop, Salvigenin, Sitosterol, Stigmasterol-beta-d-glucoside. | 2 |
| (2R)-7-hydroxy-5-methoxy-2-phenylchroman-4-one, (E)-4-Phenyl-3-Buten-2-One, 1-(4-Hydroxy-3-Methoxyphenyl)-3,5-Octane-Diol, 1,3,5-Trihydroxyxanthone, 1,6-Dihydroxy-3,5,7-Trimethoxyxanthone, 1,8-dihydroxy-4-hydroxymethyl anthraquinone, 11,13-Eicosadienoic acid, methyl ester, 1-Phenyl-1,3-Butanedion, 2-(3-Hydroxy-4-Methoxyphenyl)-Ethyl-1-O-Î‘-L-Rhamnosyl-(1â†’3)-Î’-D-(4-Feruloyl)-Glucoside, 2-(3-hydroxy-4-methoxyphenyl)-ethyl-1-o-α-l-rhamnosyl- (1→3)-β-d-(4-feruloyl)-glucoside, 2,6,2',4'-tetrahydroxy-6’-methoxychalcone, 2,6,2',4'-Tetrahydroxy-6â€™-Methoxychalcone, 2,6,2',4'-tetrahydroxy-6'- methoxychaleone, 2',7-Dihydroxy-4',5'-Dimethoxyisoflavone, 3,4',5,7-tetrahydroxyflavone-3-l-rhamnoside, 3,4',5-trihydroxy-7-methoxy-8-isopentenyl-flavone, 3,4-Dihydroxy-6,7,3',4'-Tetramethoxyflavonol, 3,5,7,2',6'-Pentahydroxy Flavone, 3,5,7,2',6'-pentahydroxy flavonol, 3,5,7,2'6'-Pentahydroxyflavanone, 3alpha-dihydrocadambine, 3beta,15zeta,16-trihydroxy isopimaric acid, 4,5,6-Trihydroxy-Aurone, 4'-Hydroxywogonin, 4'-Hydroxywogonin,5,7-Dihydroxy-8- Methoxylflavone, 5,2',5'-Trihydroxy-6,7,8-Trimethoxyflavone, 5,2',6'-Trihydroxy-7,8-Dimethoxyflavone, 5,2'-Dihydroxy-6,7,8-Trimethoxyflavone, 5,6,3',4'-tetrahydroxy-7-methoxyflavone, 5,6,7,3',4'-Pentahydroxy-3-Methoxyflavone, 5,7,2',3'-Tetrahydroxyflavone, 5,7,2',5'-Tetrahydroxy-8,6'-Dimethoxy Flavone, 5,7,2,5-tetrahydroxy-8,6-dimethoxyflavone, 5,7,2',6'-Tetrahydroxyflavone, 5,7,2'-Trihydroxy-6-Methoxyflavone, 5,7,2'-Trihydroxy-8,6'- Dimethoxyflavone, 5,7,2'-Trihydroxyflavone, 5,7,3',4'-Tetrahydroxy-6,8-Dimethoxy Flavone, 5,7,4'-Trihydroxy-6-C-Glucoside-8-C- Arabinoside Flavone, 5,7,4'-trihydroxy-6-c-glucoside-8-c-arabinosideflavone, 5,7,4'-Trihydroxy-6-Methoxyflavanone, 5,7,4'-trihydroxy-8-methoxyflavanone, 5,7,4'-Trihydroxy-8-Methoxyflavone, 5,7-Dihydroxy-6,8,2',3'-Tetramethoxyflavone, 5,8,2'-Trihydroxy-6,7-Dimethoxyflavone, 5,8,2'-Trihydroxy-7- Methoxyflavone, 5,8-Dihydroxy-6,7-Dimethoxyflavone, 5-hydroxy-4',7-dimethoxy-flavone, 5-Hydroxy-6,7,8,4'-Tetramethoxyflavone, Skullcapflavone I,Skullcapflavone II, 5-Hydroxy-7,8-Dimethoxyflavone, 6-C-Arabinopyranosyl-8-C-Glucopyranosyl -5,7-Dihydroxyflavone, 6-Methoxygossypol, 7,2',6'-Trihydroxy-5-Methoxychalcone, 7,2',6'-Trihydroxy-5-Methoxyflavanone, 7,2'6'-Trihydroxy-5-Methoxyflavanone, 7,3',4'-Trihydroxyflavone, 7-Methoxybaicalein, 8-C-Arabinopyranosyl-6-C-Glucopyranosyl-5,7-Dihydroxyflavone, 8-Methoxy-5-O-Glucoside Flavone, acacetin, alpha-phenylcinnamic acid nitrile, Baicalein, Baicalein-7-O-Î’-D-Glucopyranoside, baicalein-7-o-β-d-glucopyranoside, Beta-Skytanthine, bis[(2S)-2-ethylhexyl] benzene-1,2-dicarboxylate, Carthamidin, Chrysin, Chrysoeriol, coptisine, Dihydrobaicalin, Dihydrobaicalin_qt, DIHYDROOROXYLIN, Dihydrooroxylin A, Dihydroperilla Alcohol, ent-Epicatechin, epiberberine, Eriodictyol, Eriodictyol-7,3-Diglucoside, Eriodyctiol (flavanone), Isoschaftoside, Isoscutellarein, Isoscutellarein 8-O-Beta-D- Glucuronide, M-Methoxybenzaldehyde, Moslosooflavone, NEOBAICALEIN, Norwogonin, Nothosmyrnol, Oroxylin A, Oroxylin A 7-O-Glucuronide, Osladin, Panicolin, Paniculatin, Phenylbutanone-Glucoside, rivularin, Scutellarin, Scutevulin, Sebacic Acid, Skullcapflavone I, Skullcapflavone II, Supraene, Viscidulin Iii-6'-O-Î’-D-Glucopyranoside, viscidulin iii-6'-o-β-d-glucopyranoside, Wogonin, Wogonin 5-Î’-D-Glucoside, wogonin 5-β-d-glucoside, Wogonoside, Woodorien | 1 |
| ***4. Salvia miltiorrhiza* Bunge** | |
| Gamma-Sitosterol, Sitosterol, Î’-Sitosterol, β-sitosterol. | 4 |
| Stigmasterol | 3 |
| Alexandrin, Daucosterol, Caproic Acid, Eleutheroside A, Sitogluside, Strumaroside, Î’-Sitosterol-Î’-D-Glucoside, Baicalin, Daucosterol, Dauricine, Salvigenin, Succinic Acid. | 2 |
| (25R)-5Î‘-Spirostan-3Î’-Ol,Tigogenin, (2R)-3-(3,4-dihydroxyphenyl)-2-[(Z)-3-(3,4-dihydroxyphenyl)acryloyl]oxy-propionic acid, (6S)-6-(hydroxymethyl)-1,6-dimethyl-8,9-dihydro-7H-naphtho[8,7-g]benzofuran-10,11-dione, (6S)-6-hydroxy-1-methyl-6-methylol-8,9- dihydro-7H-naphtho [8,7-g]benzofuran-10,11-quinone, (6S,7R)-6,7-dihydroxy-1,6-dimethyl-8,9-dihydro-7H-naphtho[8,7-g]benzofuran-10,11-dione, (E)-3-[2-(3,4-dihydroxyphenyl)-7-hydroxy-benzofuran-4-yl]acrylic acid, (Z)-3-[2-[(E)-2-(3,4-dihydroxyphenyl)vinyl]-3,4-dihydroxy-phenyl]acrylic acid, 1,2,5,6-tetrahydrotanshinone, 1,2-dihydro-quinone Salvia, 1,2-Dihydrotanshiquinone, 1,5-Dihydroxy-3-Methylanthraquinone, 15,16-dihydrotanshinone i, 1-Hydroxytaxinine A, 1-keto-isocryptotanshinone, 1-methyl-8,9-dihydro-7H-naphtho[5,6-g]benzofuran-6,10,11-trione, 2-(4-hydroxy-3-methoxyphenyl)-5- (3-hydroxypropyl)-7-methoxy-3-benzofurancarboxaldehyde, 2-isopropyl-8-methylphenanthrene-3,4-dione, 2-Isopropyl-8-Methylphenanthrene-3,4 -Dione (R0-090680), 3,4-Dihydroxybenzoic Acid,Protocatechuic Acid, 3,4-Dihydroxybenzoicacid, 3-beta-Hydroxymethyllenetanshiquinone, 3beta-Hydroxytanshinone Iia, 3Î‘-Hydroxytanshinone Iia, 3-Î’-Hydroxymethylenetanshiquinone, 3Î’-Hydroxytanshinone Iia, 3-O-Acetyloleanolic Acid, 3α-HydroxytanshinoneⅡa, 3-β-Hydroxymethylenetanshiquinone, 3β-Hydroxytanshinone iia, 4-Methyl salicylaldehyde, 4-Methylenemiltirone, 5,6-Dihydroxy-7-isopropyl-1, 1-dimethyl-2,3-dihydrophenanthren-4-one, 5,7,4'-Trihydroxy-6,8,3'-Trimethoxyflavone,Labiatenic Acid,Rosmarinic Acid, 6-Hydroxymethyllumazin, 6-o-syringyl-8-o-acetyl shanzhiside methyl ester, Alexandrin,Daucosterol,Eleutheroside A, C09092, Caffeicacid, Carnosol, Cryptanshinone, Cryptotanshinone, Cryptoxanthin, D(+)3-13,4 hydroxy acid, Dan phenolic acid C, Dan phenolic acid D, Dan phenolic acid E, Dan phenolic acid F, Dan phenolic acid G, Dan phenolic acid H, Dan phenolic acid I, Dan shen-spiroketallactone, Danchensu, Danshen spiroketallactone, Danshenol A, Danshenol B, Danshenspiroketallactone, Danshensu, Danshensuan B, Danshenxinkun A, Danshenxinkun B, Danshenxinkun C, Danshenxinkun D, Danshexinkum A, Danshexinkum D, Dan-shexinkum d, Daphneolone, Dehydromiltirone, Dehydromorroniaglycone, Dehydrotanshinone II A, Dehydrotremetone, Deoxyneocryptotanshinone, Different tanshinone Ⅱ, Digallate, Dihydrokaranone, dihydrotanshinlactone, Dihydrotanshinone Ⅰ, Dihydrovalepotriate, Epidanshenspiroketallactone, Ethyl Lithospermate, Ferruginol, Ferulic Acid, Formyltanshinone, Four methyl dan phenolic acid F, Hesperetic Acid, Heteratisine, Hydroxy Tan, Hydroxytanshinone, Î”1-Dehydrotanshinone, Iso Cryptotanshinone, ISO Dan phenolic acid C, Isocryptotanshinone, Isocryptotanshi-none, Isocucurbitacin D, Isoferulic Acid, Isoimperatorin, Isotanshinone I, Isotanshinone II, Isotanshinone Iia, Isotanshinoneii B, Isotenulin, labiatenicacid, Lithospermate B, Lithospermic Acid, Iithospermicacid b, Iithospernic acid, Iuteolin, Magnesium Lithospermate B, manool, Methyl ester, Methyl Tanshinonate, Methylene Tanshinquinone, Methylenetanshinquinone, Methyltanshinonte, Microstegiol, Miltionone Ⅰ, Miltionone Ⅱ, Miltionone I, Miltionone Ii, Miltipolone, Miltirone, Miltirone Ⅱ, Mimengoside a, Mitirone, Monomethyl Lithospermate, Mono-O-Methylwightin, Neocrycasin a, Neocryptotanshinone, Neocryptotanshinone Ii, neotanshinone b, Neotanshinone C, Neotigogenin, Nortanshinone, Nortrachelogenin, NSC 122421, Oleanolic Acid, Oleoyl Danshenxinkun A, Oleoyl Neocryptotanshinone, oleoylneocryptotanshinone, O-Methyltaxodine, Oxalic acid, Paramiltioic Acid, poriferast-5-en-3beta-ol, Poriferasterol, Prgewaquinone A, Prolithospermic acid, Protocatechuic Acid-3-Glucoside, Protocatechuic Aldehyde, Protocatechuicaldehyde, Przewalskin a, Przewalskin b, Przewaquinone B, Przewaquinone c, Przewaquinone E, Przewaquinone f, Purple Salvia A prime, B prime, Prime amyl, Rosemary acid, Rosmarinic Acid Methyl Ester, Rosmarinicacid methyl ester, Rosmarinine, Rutin, Sal A, Salb, Salonitenolide, Salvia acid B, Salvia acid C, Salvia acid methyl ester, Salvia alcohol Ⅰ, Salvia alcohol Ⅱ, Salvia alcohol Ⅲ, Salvia diol A, Salvia diol B, Salvia diol c, Salvia lactone, Salvia new one, Salvia new one Ⅳ, Salvia phenol potassium, Salvia potassium, Salvia propionic acid, Salvia quinone methine, Salvianolic Acid A, Salvianolic Acid B, Salvianolic Acid C, Salvianolic Acid D, Salvianolic Acid E, Salvianolic Acid G, Salvianolic acid j, Salvianolicacid A, Salvianolicacid B, Salvianolicacid c, Salvianolicacid d, Salvianolicacid e, Salvianolicacid g, Salvilenone, Salvilenone Ⅰ, Salvinone, Salviol, salviolone, Samaderin A, Sclareol, Sugiol, Tan Ⅴ, Tanshinaldehyde, Tanshinaldehyde Ii, Tanshindiol A, Tanshindiol B, Tanshindiol C, Tanshinlactone, Tanshinol A, Tanshinol B, Tanshinone Ⅵ, Tanshinone I, Tanshinone Ii A, Tanshinone Ii B, Tanshinone Iia, Tanshinone Iib, Tanshinone Vi, Tanshiquinone a, Tanshiquinone B, Tanshiquinone c, Taraxanthin, Tigogenin, Ursolic Acid, Ursolic Protocatechualdehyde, Ursolicacid, Urushio iii, α-amyrin, Δ1-dehydrotanshinone. | 1 |
| ***5. Alisma plantago-aquatica* L** | |
| Choline | 3 |
| Sitosterol | 2 |
| [(1S,3R)-1-[(2R)-3,3-dimethyloxiran-2-yl]-3-[(5R,8S,9S,10S,11S,14R)-11-hydroxy-4,4,8,10,14-pentamethyl-3-oxo-1,2,5,6,7,9,11,12,15,16-decahydrocyclopenta [a]phenanthren-17-yl]butyl] acetate, 11-Deoxyalisol A, 13Î’,17Î’-Epoxyalisol A, 13Î’,17Î’-Epoxyalisol A 24-Acetate, 13β,17β-Epoxyalisola, 13β,17β-Epoxyalisola, 24-Acetate, 16β-Methoxyalisol B Monoacetate, 1-Monolinolein, 25-Anhydroalisol A 11-Acetate, 25-Anhydroalisol A 24-Acetate, 2-Furaldehyde, 2-Furancarboxylic Acid, Alismol, Alisol A, Alisol a 24-acetate, Alisol A Monoacetate, Alisol B, Alisol B Monoacetate, Alisol C, Alisol C Monoacetate, Alisol cmonoacetate, Alisol E 23-Acetate, Alisol E 24-Acetate, Alisol f, Alisol,b,23-Acetate, Alizexol A, Alizexol B, Neoalisol, Oriediterpenol, Oriediterpenoside, Orientalol A, Orientalol B, Orientalol C, Orientalol E, Orientalol F, Sulfoorientalol A, Sulfoorientalol B, Sulfoorientalol C, Sulfoorientalol D, Sulfopatrinoside I. | 1 |

**Supplementary Table 2:** Top 10 terms of molecular function of common targets.

| **Description** | **Overlap** | **Enrichment Ratio** | **Involved proteins** |
| --- | --- | --- | --- |
| signaling receptor activity | 117 | 4.474 | ABCA1; ABCA7; ABCC8; ABCC9; ADORA2A; ADORA2B; ADRA1A; ADRA2A; ADRB1; ADRB2; AGER; AR; AVPR1A; CD36; CHRM1; CHRNA2; CHRNA3; CHRNA4; CHRNA6; CHRNA7; CHRNB2; CHRNB4; DRD3; DRD4; EPHA4; ESR1; ESR2; FAS; FCER1G; GRIN2B; GRIN3A; GRIN3B; HTR2A; HTR2C; ICAM1; INSR; LHCGR; MC2R; NOTCH4; NR1H2; NR3C1; NTRK1; PPARA; PPARD; PPARG; RXRA; RXRB; RXRG; SCARB1; SEMA4D; SORCS3; SORT1; TACR2; THRA; VDR; VLDLR; FGFR1; FGFR2; KIT; KDR; AGTR1; MC4R; CHRM3; PDGFRB; MET; HTR1A; HTR1B; HTR4; DRD2; GABRA5; GRIN1; GRIN2A; CHRM2; TSPO; HTR1D; ADRA2C; GRIA1; PTGFR; RARB; SSTR2; SSTR5; GABRG3; CHRM5; FGFR3; RARA; HRH3; HTR3A; MST1R; F2; GRIN2C; GRIN2D; GABRA1; GABRA2; GABRA3; GABRA4; GABRA6; GABRB1; GABRB2; GABRB3; GABRD; GABRE; GABRG1; GABRG2; GABRP; GABRQ; GLRA1; GLRA2; GLRA3; GLRA4; GLRB; DRD1; CHRM4; ADRA1B; DRD5; ADRB3; ADRA1D; ADRA2B. |
| protein dimerization activity | 75 | 3.227 | ABCG1; ABCG4; ACHE; ADORA2A; ADRA1A; ADRA2A; ADRB1; ADRB2; AHR; APOA2; APOC2; APOE; AR; BCL2; CAMK2G; CAT; CAV1; CCL5; CHRNA7; CHRNB2; CHRNB4; CSF1; ECE1; ENPP1; FCER1G; FLOT1; FOS; GSTM1; HIF1A; HMGCR; HMOX1; LRRK2; MAOB; NCOA2; NOTCH4; NTRK1; PIK3R1; PON1; PPARD; PPARG; PPP3CB; PTGS2; RXRA; SCARB1; SERPINF2; SLC11A1; SLC6A4; SMAD3; SREBF1; SUPV3L1; TGFB1; TP53; WRN; FGFR1; FGFR2; KIT; AGTR1; HSP90AA1; DRD2; ADRA2C; NFKB1; RELA; VEGFA; TOP2A; IKBKB; RARA; FLNA; BAX; TPI1; CTNNB1; NOS2; SOD1; ADRA1B; ADRB3; KCNH2. |
| ion channel activity | 49 | 6.453 | ABCC8; ABCC9; CACNB2; CALHM1; CHRNA2; CHRNA3; CHRNA4; CHRNA6; CHRNA7; CHRNB2; CHRNB4; GRIN2B; GRIN3A; GRIN3B; KCNJ6; KCNMA1; HTR1B; GABRA5; GRIN1; GRIN2A; SCN5A; GRIA1; CACNA1C; GABRG3; CACNA1D; KCNN4; GRIN2C; GRIN2D; HTR3A; GABRA1; GABRA2; GABRA3; GABRA4; GABRA6; GABRB1; GABRB2; GABRB3; GABRD; GABRE; GABRG1; GABRG2; GABRP; GABRQ; GLRA1; GLRA2; GLRA3; GLRA4; GLRB; KCNH2. |
| anion channel activity | 21 | 13.191 | GABRA5; GABRG3; GABRA1; GABRA2; GABRA3; GABRA4; GABRA6; GABRB1; GABRB2; GABRB3; GABRD; GABRE; GABRG1; GABRG2; GABRP; GABRQ; GLRA1; GLRA2; GLRA3; GLRA4; GLRB. |
| transmitter-gated ion channel activity | 35 | 31.356 | CHRNA2; CHRNA3; CHRNA4; CHRNA6; CHRNA7; CHRNB2; CHRNB4; GRIN2B; GRIN3A; GRIN3B; GABRA5; GRIN1; GRIN2A; GRIA1; GABRG3; GRIN2C; GRIN2D; HTR3A; GABRA1; GABRA2; GABRA3; GABRA4; GABRA6; GABRB1; GABRB2; GABRB3; GABRD; GABRE; GABRG1; GABRG2; GLRA1; GLRA2; GLRA3; GLRA4; GLRB. |
| carboxylic acid binding | 27 | 7.341 | ALB; ALOX5AP; DPYS; GOT1; GRIN2B; GRIN3A; GRIN3B; LRAT; NOS1; NOS3; OTC; PCK1; PLA2G1B; PPARA; PPARD; PPARG; RXRA; SNCA; VDR; GRIN1; RARA; NOS2; GLRA1; GLRA2; GLRA3; GLRA4; GLRB. |
| serotonin receptor activity | 13 | 21.528 | CHRM1; DRD4; HTR2A; HTR2C; CHRM3; HTR1A; HTR1B; HTR4; CHRM2; HTR1D; HRH3; CHRM4; CHRM5. |
| peptide binding | 30 | 5.596 | ACHE; ADRB2; AGER; APOA1; APOE; AVPR1A; BCHE; CD36; CHRNA7; CLU; CRHBP; ECE1; EPHA4; GRIN2B; GSTM1; GSTP1; INSR; MME; NFKBIA; PIK3R1; PPARG; RXRA; SCARB1; MC4R; GRIN1; GRIN2A; GRIA1; SSTR2; SSTR5; PTGES. |
| steroid hormone receptor activity | 15 | 15.467 | AR; ESR1; ESR2; NR1H2; NR3C1; PPARA; PPARD; PPARG; RXRA; RXRB; RXRG; THRA; VDR; RARA; RARB. |
| acetylcholine binding | 9 | 28.932 | ACHE; CHRNA2; CHRNA3; CHRNA4; CHRNA6; CHRNA7; CHRNB2; CHRNB4; CHRM3. |

**Supplementary Table 3**: Top 10 terms of KEGG pathway of common targets.

| **Gene Set** | **Description** | **Size** | **Expect** | **Ratio** | **P Value** | **FDR** |
| --- | --- | --- | --- | --- | --- | --- |
| GO:0050786 | RAGE receptor binding | 11 | 0.018 | 168.360 | 6.20E-07 | 1.16E-03 |
| GO:0050544 | arachidonic acid binding | 5 | 0.008 | 246.930 | 2.52E-05 | 1.42E-02 |
| GO:0035662 | Toll-like receptor 4 binding | 5 | 0.008 | 246.930 | 2.52E-05 | 1.42E-02 |
| GO:0050542 | icosanoid binding | 6 | 0.010 | 205.780 | 3.78E-05 | 1.42E-02 |
| GO:0050543 | icosatetraenoic acid binding | 6 | 0.010 | 205.780 | 3.78E-05 | 1.42E-02 |
| GO:0035325 | Toll-like receptor binding | 12 | 0.019 | 102.890 | 1.65E-04 | 5.17E-02 |
| GO:0036041 | long-chain fatty acid binding | 13 | 0.021 | 94.974 | 1.95E-04 | 5.23E-02 |
| GO:0043177 | organic acid binding | 212 | 0.343 | 11.648 | 3.55E-04 | 8.33E-02 |
| GO:0008017 | microtubule binding | 239 | 0.387 | 10.332 | 5.58E-04 | 1.16E-01 |
| GO:1901567 | fatty acid derivative binding | 27 | 0.044 | 45.728 | 8.65E-04 | 1.62E-01 |
